# Modality Matters: Fasted Individuals Inhibit Food Stimuli Better Than Neutral Stimuli for Words, but Not for Pictures

**DOI:** 10.3390/nu16142190

**Published:** 2024-07-09

**Authors:** Mechteld M. van den Hoek Ostende, Ulrike Schwarz, Caterina Gawrilow, Barbara Kaup, Jennifer Svaldi

**Affiliations:** Department of Psychology, Faculty of Science, University of Tuebingen, Schleichstr. 4, 72076 Tuebingen, Germany; ulrike.schwarz@johanniter.de (U.S.); caterina.gawrilow@uni-tuebingen.de (C.G.); barbara.kaup@uni-tuebingen.de (B.K.); jennifer.svaldi@uni-tuebingen.de (J.S.)

**Keywords:** homeostasis, satiation, cognitive control

## Abstract

The current study aimed to evaluate the effect different modalities (pictures and words) of food stimuli have on inhibitory control under different homeostatic states. To this end, the homeostatic state was altered by asking participants to fast for 16 h (*n* = 67) or eat lunch as usual (*n* = 76) before completing an online stop-signal task with modal (pictures) and amodal (words) food and valenced-matched non-food stimuli. The inclusion of non-food stimuli allowed us to test the food specificity of the effect. We found a significant Group × Modality × Stimulus Type interaction (*F*(1,141) = 5.29, *p* = 0.023, η_p_^2^ = 0.036): fasted individuals had similar inhibitory capacity for modal and amodal food stimuli but better inhibitory capacity for non-food words compared to images, while there were no inhibitory differences in dependence on either modality or stimulus type in satiated individuals. Thus, we were able to show that inhibitory capacities to modal compared to amodal stimuli depend on participants’ current state of fasting. Future studies should focus on how this lowered inhibitory capacity influences food intake, as well as the role of stimulus valence in cognitive processing, to clarify potential implications for dieting and weight loss training.

## 1. Introduction

The social infrastructure of Western societies contributes to the overconsumption of food of little nutritional value and fosters overweight and obesity [1]. In this diverse environment, food cues take many different forms. At a distance, abstract cues such as signs and menus can be observed, whereas sensory cues become more salient in higher proximity to food when we can experience their smell, sight, and, potentially, taste. Repeatedly resisting cravings induced by food cues becomes more difficult in the fasting state when these cues become more salient [2]. Indeed, exposure to food cues when fasting can increase food approach behavior; those with lower impulse control will select more products when fasting than when satiated [3]. Thus, reducing food intake to counteract overeating and weight gain may exacerbate impaired control over food intake by inducing hunger, ironically promoting uncontrolled eating and hindering individuals in their attempts to lose weight. A thorough understanding of the altered food processing in the fasting state is therefore paramount to combat the physical and mental consequences of overeating.

According to the reflective-impulsive model [4], non-homeostatic eating (i.e., eating in the absence of hunger) can be ascribed to a dual cognitive impairment. On the one hand, excessive impulsive processing capitalizes on food cues in our environment, exaggerating the attentional and motivational resources assigned to food consumption. On the other hand, exhausted resources of the reflective system result in lowered cognitive control, which hinders efforts to inhibit consumption. Notably, these tendencies are enhanced under relevant motivational states, such as hunger [4,5]. Furthermore, hunger is linked to enhanced anticipatory and consummatory reward responses [6,7]. Thus, hunger is associated with altered cognitive and physiological processes that are adaptive for homeostatic (i.e., hunger-driven) eating. However, these same processes can contribute to non-homeostatic eating, which puts people at risk for overweight and obesity [8,9]. Altered food cue processing during hunger may, therefore, be analogous to altered processes in individuals that are prone to non-homeostatic eating. Thus, developing a better understanding of the cognitive changes that promote homeostatic eating also provides insight into relevant pathways for non-homeostatic eating. As decreased inhibitory control is also associated with increased food intake [10,11], the relationship between inhibitory control and food deprivation may be a particularly promising avenue for understanding cognitive adaptations under fasting conditions that promote food intake.

Still, the relationship between aberrant inhibitory control and food deprivation remains ambiguous. Hunger, as an internal state, may influence inhibitory control with regard to state relevant cues, such as food cues (Jones et al., 2018) [12]. In studies using go/no-go tasks as a measure of inhibitory control, researchers found evidence of decreased inhibitory capacity to food stimuli within hungry participants, either when paired with non-food stimuli [13] or when high- and low-caloric stimuli were compared [14]. Likewise, Bartholdy et al. [15] found that fasted individuals engaged in steeper delay discounting, another marker of lowered inhibitory control, which quantifies the preference for small immediate rewards over large future rewards. Paradoxically, within the same study, they additionally found that fasting improved participants’ inhibitory control on a stop-signal task [16,17]. This result needs to be interpreted with caution, however, since too few trials were included in the paradigm for a reliable estimate of inhibitory control [18]. Thus, there are indications for reduced inhibitory capacity in fasted individuals, but the issue warrants further research.

At present, it is unknown which food cue modalities elicit the need for inhibitory control. Several studies have shown that exposure to food cues in various modalities can lead to increased caloric intake [19,20,21] and that motivational responses are enhanced under food deprivation [6,22]. This suggests a general need for inhibitory control when encountering food cues. Specifically, olfactory [19,20,21], visual, and cognitive [19] food cues have been shown to influence food intake, hunger, or food craving [22]. However, as of yet, researchers in this domain have not focused on the difference between different stimulus modalities, such as words vs. pictures, which might give rise to different types of representations and, therefore, differentially affect inhibitory control (see e.g., [23], dual-coding theory). Pictures of objects share more of the relevant sensory properties with the objects they depict and may thus activate more modal representations compared to words referring to these objects, which might be associated with more amodal representations (for a discussion, see [24]). Indeed, as part of their theory, Strack and Deutsch [4] posit that the two postulated systems operate on different representations. Whereas the impulsive system operates through behavioral schemata activated by associated input from the environment, the reflective system processes semantic and episodic information to arrive at behavioral intentions. Therefore, stimuli with different degrees of sensory properties may be preferentially processed by different systems. Specifically, modal (sensory) stimuli have an immediate relevance for behavioral schemata pertaining to eating. This type of information processing is associated with impulsive processing. Indeed, this is in line with other areas of research [25]. For example, it has been shown that pictorial negations ease the execution of the corresponding behavior [26] and more strongly activate affective processes [27]. The latter is particularly interesting, as increased arousal to food cues may contribute to overeating [28,29]. Amodal (abstract) stimuli, on the other hand, do not have immediate implications for behavior and may rather speak to the evaluative semantic processes of the reflective system. If true, in a fasted state, in which behavioral schemata pertaining to food intake are highly relevant, we would expect modal stimuli to directly activate the impulsive system, bringing on the abovementioned impaired inhibitory control. Amodal stimuli, on the other hand, should not have as large an impact since they only influence impulsive tendencies indirectly over the reflective system. In contrast to these considerations, a recent review that took into account stimulus modality (pictures vs. words) observed an effect of stimulus type for alcohol but not for food stimuli [12]. However, this review was based on studies using a range of paradigms for testing inhibitory control, making their results less comparable. Moreover, none of these paradigms directly compared various modalities for food stimuli.

Given the importance of this factor for the impulsive-reflective model (see above), the current study aims to examine multiple food cue modalities in a single paradigm in order to obtain a more precise estimate of their effects. Because we conceptualize modality on a spectrum from sensory to abstract [25] and the sensory input should remain consistent, we used (non)-food pictures as modal stimuli and (non)-food words as amodal stimuli. As valence could influence SST performance [30,31], we included valenced-matched control stimuli (animals) to determine whether the effects would be food-specific. Notably, hunger modulates food processing but not the processing of high-valence pictures [32]. The aim of this study was to evaluate the effect of food cues in different modalities (pictures and words) on inhibitory control under different homeostatic states (fasting and satiated). Observed differences in inhibitory control specific to food cues in either modality would underscore the need for a better understanding of how different food cues influence food intake. To this end, we conducted a pre-registered online study during the COVID-19 pandemic. Fasted and satiated participants completed a stop-signal task (SST [16,17]) as an inhibitory control task with modal (images) and amodal (words) food and non-food cues. In line with previous research [13,14], we expected longer SSRTs (thus reduced inhibitory control) for fasted compared to satiated participants for modal food stimuli, while we did not expect to find this difference for modal non-food stimuli. We expected no significant differences in the SSRTs between food and non-food amodal stimuli. We also did not expect the fasted and satiated groups to have different SSRTs for amodal stimuli.

## 2. Materials and Methods

This study’s hypotheses, planned sample, variables, and paradigms were preregistered and can be found on the Open Science Framework (https://osf.io/vk7he (accessed on 3 June 2024), with data and analysis available at https://osf.io/472kr/ (accessed on 3 June 2024). This study ran from mid-April until the end of May 2022.

### 2.1. Participants

Participants were recruited using Prolific (www.prolific.co (accessed on 3 June 2024)). Inclusion and exclusion criteria were enforced through a pre-study screening questionnaire. Exclusion criteria were current pregnancy or lactation, bad eyesight, and color blindness. Additionally, participants were excluded if they were unable to fast due to expected/potential physical (e.g., diabetes mellitus) or mental (e.g., eating disorders) negative effects or if they were unwilling to do so for other reasons. Vegan nutrition was a further exclusion criterion due to the use of a stimulus set that included non-vegan products (e.g., cheese). As hunger modulates the processing of food cues in normal weight but not overweight participants [9,13], included participants needed to have a body mass index (BMI) between 18.5 and 25 kg/m^2^. Participants also had to be between 18 years and 30 years old and speak English as their first language. Participants were compensated a total of £10.25 for completing this study. Partial credit was given to participants who only completed the pre-selection questionnaire.

The sample size was calculated using a priori power analysis through Superpower [33]. Means and standard deviations for modal and amodal food stimuli were taken from a pilot study. With a power of (1 − β) = 0.90, α = 0.05, we required 159 participants per group, totaling 318 participants. Given a dropout rate of 15% due to SST performance incompatible with the horse-race model [18], we aimed to recruit 366 individuals (i.e., 183 fasted and 183 satiated). A total of 367 participants completed the full study, out of which 164 were discarded: 31 participants did not fill out one or more of the questionnaires, 71 demonstrated behavior on the SST that was incompatible with the horse-race model [18], and out of the remaining participants, 122 did not fulfill the fasted or satiated criteria (see 2.1.1). We thank an anonymous reviewer for suggesting the use of stringent inclusion criteria, leaving a total sample of 143 participants (67 fasted and 76 satiated). This reduced our power to only (1 − β) = 0.55 for the expected effect size.

### 2.2. Design and Procedure

This study required participation in two parts, both completed fully online and on separate days. In the first step (10 min), participants completed a screening of inclusion and exclusion criteria via the SoSci Survey [34]. At the end, they were instructed to choose a day in the upcoming week in which they could either fast overnight or eat their meals as usual, depending on random assignment. On this day, they needed to complete the second part of this study through a personalized link either before (fasted condition) or after (satiation condition) lunch.

On this second day (approximately 50 min, depending on variation in reaction times in the SST and questionnaire completion times), participants indicated their hunger levels among filler state questions on a 0–100 scale visual analogue scale (VAS), after which they completed the questionnaires and the SST. This included 16 practice trials in which participants could familiarize themselves with the task. Practice trials included a random subset of stimuli from both modal (picture) and amodal (word) categories. Finally, participants indicated when they last consumed calories under the assurance that this would not influence their compensation.

### 2.3. Stop-Signal Task

Participants indicated the location of continuously presented words or pictures by key press [35]. In some trials, a stop signal followed the presentation of the stimulus. Participants were instructed to inhibit their response upon seeing this stop signal. The task ran with two separate sets of picture and word stimuli so that the pictures of the first set were described using the words of the second set and vice versa. Participants were randomly assigned a stimulus set at the beginning of the SST. Overall, we had 32 pictures of high-caloric food and 32 non-food pictures (animals). Pictures were complexity-matched within their set using the ‘imagefluency’ package in R [36]. For the amodal stimuli, we selected 32 corresponding English food words and 32 non-food (animal) words. Words were matched per set in length and frequency using the Corpus of Contemporary American English [37]. This corpus represents word frequencies in American media such as film, print, and webpages. Pictures were pre-rated and taken from our internal database.

**Figure 1:**
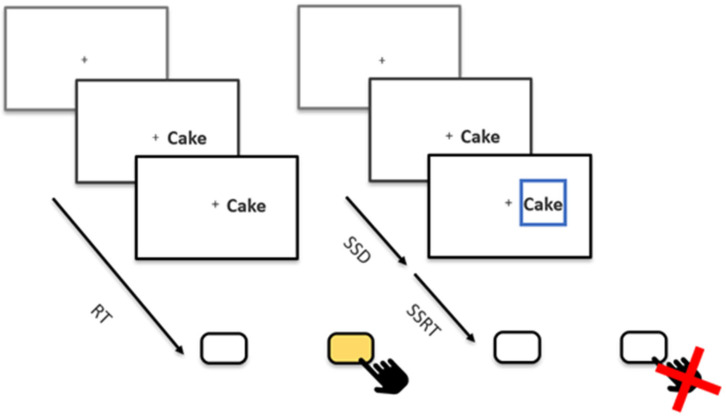
A Schematic Overview of the Stop-Signal Task. The fixation cross was presented for 250 ms, followed by the stimulus presentation until participants responded or for a maximum of 1250 ms. No feedback was given between trials; instead, participants received feedback on their performance during the 7 breaks between blocks. Each break lasted 15 s. The initial SSD was set to 200 ms, which was shortened (missed) and lengthened (stopped) by 50 ms after each stop trial. RT = Reaction Time, SSD = Stop-Signal Delay, SSRT = Stop-Signal Reaction Time.

Besides 16 practice trials, participants completed a total of eight blocks of 128 trials each, totaling 1024 trials. In 25% of trials, the stop signal was shown. The stop-signal presentation was balanced over blocks and over stimulus types. All trials started with a fixation cross, which was displayed for 250 ms. Afterwards, a food picture or word was presented to either the left or right of the fixation cross. Participants were instructed to indicate whether the stimulus appeared left or right of a fixation cross with the right and left arrow keys as quickly and accurately as they could (Figure 1). Stimuli remained on the screen for up to 1250 ms or until a response key was pressed. The modality of the stimulus and its content were irrelevant to the task. On stop trials, a stop signal in the form of a blue box appeared around the stimulus with a variable delay after stimulus onset. This stop-signal delay (SSD) was originally set to 200 ms and increased or decreased depending on the individual participant’s performance. If participants correctly inhibited their response, the SSD was increased by 50 ms, making stopping on subsequent trials more difficult. If, on the other hand, participants incorrectly pressed a key, the SSD was decreased by 50 ms. The goal of this tracking algorithm was that the participant could inhibit around 50% of all stop trials, which is required for a reliable calculation of the Stop-Signal Reaction Time (SSRT [18]), our imputed measure of inhibitory control. SSDs tracked for all combinations of modality and stimulus type (picture–food, picture–non-food, word–food, and word–non-food) to later estimate independent SSRTs.

To ensure limited interactions between pictures and words, each block exclusively contained trials of one of these modalities. Blocks alternated between modalities, and the starting block was counterbalanced. Between each block, participants took 60-s breaks, during which they received feedback on their performance; they were presented with their (overall) mean reaction time, the number of go-trial omissions, and the percentage of successfully inhibited stop-trials, together with a reminder that go-trial omissions should be 0 and that around 50% of stop-trials should be successfully inhibited [18]. Participants were instructed not to wait for the stop signal. The SST was programmed with jsPsych [38] and an adaptation of the STOP-IT paradigm [18]. Previous versions of the SST demonstrated poor to good test–retest reliability [39,40,41], while models indicate higher reliability of SSRT estimates for the integration method used here [18].

### 2.4. Questionnaires

To assess participants’ eating disorder pathology, participants completed the Eating Disorder Examination Questionnaire (EDE-Q [42]), which consists of 28 items that are answered on a 7-point Likert scale. To assess the severity of depressive symptoms, participants completed the Beck Depression Inventory-II (BDI-II [43]), a self-report measure that consists of 21 items rated on 0–3 Likert scales. Both the EDE-Q [44] and the BDI-II [45] have good psychometric properties. In the present study, Cronbach’s α was α = 0.94 for the EDE-Q and α = 0.92 for the BDI-II. Additionally, participants completed the Restraint Scale (RS [46]). The RS was added for exploratory purposes since restraint eating is our grouping variable for a related project. The scale has 10 items, which reflect weight fluctuations and diet attempts. Cronbach’s alpha for the RS in the present sample was α = 0.81.

### 2.5. Data Analysis

All data were analyzed in R [47] using the tidyverse package [48]. We pre-processed and calculated modality-specific SSRTs using the integration method with the replacement of response omissions [18]. This method allows for a reliable estimate of the SSRT since it accounts for omitted go trials and incorporates every individual’s stopping probability [18]. We subjected reaction times and SSRT estimates to a 2 (Group: fasted vs. satiated) × 2 (Modality: picture vs. word) × 2 (Stimulus type: food vs. non-food) mixed-model analysis of variance (ANOVA). Effect sizes are reported as partial eta-squared (η_p_^2^) and are defined as small (η_p_^2^ = 0.01), medium (η_p_^2^ = 0.06), and large (η_p_^2^ = 0.14) [49].

## 3. Results

### 3.1. Group Characteristics

Fasted and satiated groups did not differ significantly in terms of sex distribution, age, body mass index (BMI), BDI-II score, or EDE-Q (sub)scores. Crucially, groups significantly differed in their self-reported hunger, with the fasted group reporting higher hunger levels than the satiated group (Table 1).

### 3.2. Stop-Signal Reaction Time

**Figure 2:**
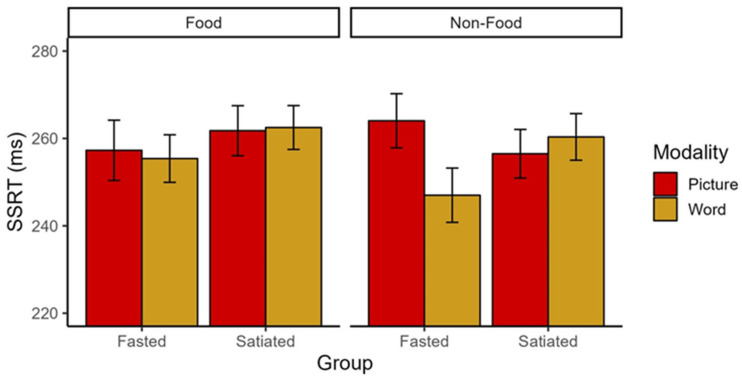
SSRT for Group, Modality, and Stimulus type. Individuals who are fasted have better inhibitory capacity for non-food words compared to pictures, whereas individuals who are satiated do not show this tendency. The figure reflects mean and standard errors. SSRT = Stop-Signal Reaction Time.

We observed a significant interaction of Group × Modality × Stimulus Type of small to medium effect size (*F*(1,141) = 5.29, *p* = 0.023, η_p_^2^ = 0.036; Figure 2) in the absence of main effects (*F*s < 1.75, *p*s > 0.19). Post-hoc 2 (Modality: picture vs. word) × 2 (Stimulus type: food vs. non-food) repeated measures ANOVAs conducted separately for each group revealed a significant interaction of Modality × Stimulus Type with a medium to large effect size (*F*(1,66) = 8.20, *p* = 0.006, η_p_^2^ = 0.11) and a main effect of Modality of medium effect size (*F*(1,66) = 5.21, *p* = 0.026, η_p_^2^ = 0.07) in the fasted group. As expected, there were no significant differences in the SSRT in the satiated group (all *F*s < 1.44, *p*s > 0.23). Against our expectations, follow-up *t*-tests revealed that fasted participants had a lower SSRT for words compared to pictures in the non-food condition (*t* (66) = 10.25, *p* = 0.002), indicating better inhibitory control for words (Figure 2). In the food condition, there were no SSRT differences between words and pictures (*t* (66) = 0.177, *p* = 0.675). There were no differences between stimulus types within the picture or word modality (all *t*s < 3.00, *p*s > 0.088).

### 3.3. Reaction Time

We found a significant main effect of Modality on reaction time (*F*(1,141) = 17.64, *p* < 0.001) so that participants reacted faster to words than to images. We further found a main effect of Stimulus Type (*F*(1,141) = 24.73, *p* < 0.001), with participants reacting quicker to non-food compared to food stimuli. There was also a significant interaction of Group × Stimulus Type (*F*(1,141) = 4.25, *p* = 0.039), with satiated participants reacting quicker than fasted participants in the non-food condition but not in the food condition. There were no other significant main effects or interactions (all *F*s < 3.36, *p*s > 0.070). Average reaction times, as well as other SST parameters, can be found in the Supplementary Materials (Table S1).

## 4. Discussion

The reflective-impulsive model [4] is often used to explain the decreased inhibition for salient food cues observed in heightened motivational states, such as hunger or aberrant eating behavior. However, the influence of different types of mental representations on these processes has been largely neglected. Here, to better understand how inhibitory control interacts with regular homeostatic appetite regulation, we used sensory, modal (picture), abstract, amodal (word) food, and non-food stimuli in a common inhibition paradigm to test their impact on fasted and satiated individuals. We assumed that pictures would be associated with more modal representations while words would be associated with more amodal representations of the respective content. Crucially, we found an interaction that points to an inhibitory advantage specifically for amodal over modal non-food stimuli in fasted individuals, which could not observed in satiated individuals. Therefore, our results indicate a hitherto unrecognized interaction between mental representations and homeostasis in inhibitory control.

Still, the direction of these processes did not align with our expectations. Originally, we postulated that modal food stimuli would primarily activate the impulsive system, which should correspond to inhibitory difficulties for food after fasting when it is highly relevant. Conversely, we expected that amodal stimuli would activate the reflective system, thus omitting the activation of approach tendencies and the associated reduced inhibitory capacity in both fasted and satiated states. However, in line with Bartholdy, Cheng, Schmidt, Campbell, and O’Daly [15], we did not find any differences between groups or modalities in the food condition. Rather, we found an inhibitory advantage for amodal over modal stimuli for non-food stimuli in fasted individuals. As such, potentially amodal stimuli only weaken inhibitory control if they are state-relevant (i.e., food stimuli in the current study), whereas state-irrelevant amodal stimuli are associated with better inhibition. Because decreased inhibitory control is associated with increased food intake [10,11], understanding which stimuli elicit decreased inhibitory control may help us target specific stimuli when designing training programs to increase inhibitory control. Indeed, training programs that repeatedly require the inhibition of tempting food stimuli can reduce subsequent food intake [50,51], and understanding which stimuli are particularly threatening under conditions of satiety and hunger may help us to improve these training programs.

Paradoxically, however, we found no differences in inhibitory control in satiated individuals. This may be due to the nature of the non-food stimuli. These were valence-matched rather than neutral to eliminate valence as a possible explanatory variable of our results. However, this positive valence may have differentially affected inhibitory processes. In alcohol-dependent individuals, for instance, word valence influenced stopping processes independent of cue exposure [30]. In a healthy sample, valenced pictures have also affected stopping efficiency [31]. As the food stimuli did not carry an additional motivational relevance for the satiated group, the food stimuli may have elicited different inhibitory processes in satiated individuals, potentially explaining why we only find modality and stimulus-dependent differences in the fasted group. Our reaction time data may even indicate that food had a lower valence in the satiated group. In the present study, satiated individuals displayed faster reaction times only to non-food stimuli compared to fasted participants. Assuming that faster reaction times indicate increased attentional allocation and stimulus relevance [52], it is possible that non-food stimuli had a higher valence in the satiated group. Thus, the interaction between Group and Stimulus Type in the present study may reflect an unexpected discrepancy in valence in satiated individuals. This discrepancy may be due to the fact that the ratings used for stimulus selection were obtained from participants in different states of satiety, and food stimuli have less relevance when satiated. To further investigate how valence influences performance in both reaction time and SSRT, future studies should, therefore, use stimulus types with varying degrees of valence and include concurrent stimulus valence ratings. It should also be noted that, due to our significantly reduced power, our null results must be interpreted with caution, as smaller effects may be masked. Despite this limitation, we can observe that even if small true effects are present in the data, they are not consistent with our hypotheses: in all conditions except for non-food pictures, the fasted group had descriptively better inhibitory control than the satiated group, whereas we expected the fasted group to have worse inhibitory control in the modal food condition. Therefore, the current data seem to support a rejection of the original hypotheses.

Nevertheless, the current results provide a first indication that stimulus modality cannot be assumed to be trivial regarding inhibitory control. This extends previous research, which also found that modality can affect performance in a dot-probe task, albeit as a function of caloric value rather than the homeostatic state [53]. Thus, the choice of stimulus modality in food processing studies may be a source of heterogeneity in the existing literature. Moreover, stimulus modality can be considered a potential factor of interest in abnormal eating due to its interaction with the homeostatic state. In fact, one study has shown that hunger interacts with reduced inhibitory control to induce higher caloric intake [3]. Others have found that hunger differentially affects inhibitory control and reward sensitivity in individuals with overweight [54] and unsuccessful dieters [55]. Future studies should, therefore, consider trait-like eating behavior and weight factors when further investigating the interaction between stimulus modality and homeostasis. Furthermore, although addressing more amodal stimuli already expands the range of relevant stimuli, previous studies have shown that other sensory stimuli, such as olfactory food cues [19,20,21], and abstract stimuli, such as cognitive food cues [19], also induce hunger and craving. If we conceptualize modality as a spectrum [25], with food pictures and smells on the modal side and words and cognitions on the amodal side of the spectrum, we might expect inhibitory control over olfactory stimuli to mimic food pictures, whereas cognitive stimuli might mimic our results related to food words. In addition, the combination of multiple sensory inputs may influence our perception and intake of food [56], but their effects on inhibitory control remain unexplored. Finally, in accordance with reduced inhibitory control in individuals with overweight [57] or unsuccessful dieters [58], training programs have been developed to improve food-related inhibition in these groups [59]. However, these training programs have mostly targeted pictures (e.g., [60,61]), whereas our results suggest that words similarly elicit inhibitory control. Thus, future inhibition training programs may benefit from incorporating a variety of stimuli and contexts that better reflect the diversity of sensory and abstract experiences in our food environment.

This study has several limitations. First, due to unexpectedly high dropout rates, our sample size was smaller than required to achieve the intended statistical power. Notably, though, this limited power specifically decreased our ability to find the assumed three-way interaction. Since the identified three-way interaction differed from our initial assumptions and the descriptive direction of the effects in the data contradicts our initial hypothesis, we still have grounds to reject this initial hypothesis. Therefore, replication of this unexpected effect in a laboratory setting is crucial for consolidating these findings. Furthermore, because previous laboratory SST experiments have shown lower attrition rates (e.g., [57,62]), such studies are needed to control for any selection bias introduced by administering the stop-signal task online rather than in the laboratory. Second, our stimuli were irrelevant to the task. To categorize the images, participants only had to indicate the location of the stimulus appearance. Also, as we did not ask participants to freely recall or identify the stimuli presented, we do not know the extent to which participants processed pictorial and verbal stimuli. As such, the depth of processing may have influenced the effect of the stimuli on cognitive control [62]. In particular, since such processing would enhance modality differences, the presence of an interaction between stimulus modality and homeostasis in a task that did not require stimulus processing highlights its potential relevance. Third, as mentioned above, based on pilot pre-ratings of the stimuli used, our animal stimuli were positive in valence rather than neutral, which may have influenced both attentional [63] and inhibitory processing [31]. Therefore, image valence should be assessed concurrently to test for valence as a possible moderator. Fourth, the word and picture stimuli had distinct visual properties; where the pictures had a range of colors, the words consisted of simple black and white letters, which were exclusively in English. It is, therefore, possible that the effects we find are based on these different visual properties rather than the processing of their meaning or using different languages or writing systems. Hence, future studies should control for these differences by manipulating meaning in addition to valence, e.g., by employing illegible scripts. Similarly, order effects on the SSRT could indicate that the representations of pictures and words influence each other, even when a one-to-one pairing is not possible and stimuli are divided into pictures and words based on subcategories (sweet and savory). Further research is therefore needed to better understand how these representations affect inhibition individually and how they interact when used together. Fifth, it is likely that our study suffered from selection bias. Although dropout rates were similar for fasted and satiated individuals, participants were explicitly asked whether they were theoretically willing to be assigned to either group. Those who indicated they would not accept their randomized assignment were not invited for further participation. Hence, the current study does not represent participants who are theoretically unwilling to fast. Furthermore, because hunger affects food processing differently in individuals who are overweight [9], we included only individuals with normal weight in the current sample. Therefore, our results are limited to altered processing under different homeostatic states in a population that has relatively stable homeostatic control over its food intake. Because the processes that influence homeostatic and nonhomeostatic eating overlap at several nodes in the brain’s feeding and reward systems [8], the current findings can only be used to hypothesize that modality may differentially affect the processing of food cues in populations characterized by nonhomeostatic eating. Sixth, it is important to acknowledge that the current experiment only sheds light on inhibitory control as measured via the SST. Paradigms such as the go/no-go task measure other aspects of inhibitory control [64], so further research is needed to fully understand the impact of modality on this overarching construct. Finally, because the studies were conducted entirely online due to the COVID-19 pandemic, we had no control over actual food intake, the device used to complete the SST, or other confounding factors such as sleep, medication use, stress, and emotional state. Although we instructed participants to complete the task in a distraction-free location, the (food) environments in which participants completed the paradigm were also likely to be highly variable. This variability also prevented us from using more objective measures of eating behavior, which we attempted to overcome by asking participants to report their last meal. However, self-reported food intake is thought to be inaccurate with respect to caloric intake [65]. As we attempted to systematically manipulate hunger, variation in reported intake may have influenced participant inclusion and exclusion criteria for participants, thereby exacerbating our selection bias. We must, therefore, expect increased noise in our data, highlighting the importance of controlled laboratory studies to replicate these findings, especially when controlling for hunger levels.

## 5. Conclusions

In summary, we found evidence that homeostatic state, stimulus type, and stimulus modality interact in their influence on inhibitory control over positively valenced stimuli. Specifically, whereas fasted individuals have better cognitive control for words compared to pictures, no such differences are found in satiated individuals. A deeper understanding of these processes may help us to better understand how the homeostatic state interacts with the food environment to drive eating behavior, which, in turn, has implications for weight regulation through restrictive dieting. Further research is needed to better understand the mechanisms underlying this relationship, particularly with regard to affective processing and its implications for populations who struggle to control food intake.

## Figures and Tables

**Table 1:** Group characteristics. BMI = body mass index, BDI-II = Beck’s Depression Inventory-II, EDE-Q = Eating Disorder Examination Questionnaire, RS = Restraint Scale.

	Fasted Mean (SD)	Satiated Mean (SD)	Statistics	*p*-Value
*n*	67	76		
Sex (n women)	43	55	χ^2^(1) = 1.59	0.21
Age	24.3 (3.9)	24.9 (3.4)	*t*(141) = −1.05	0.30
Hunger	72.1 (23.3)	16.3 (16.3)	*t*(141) = 18.3	<0.001
BMI	21.9 (1.7)	21.6 (1.9)	*t*(141) = 0.97	0.34
RS	11.9 (6.2)	11.6 (5.9)	*t*(141) = 0.25	0.81
EDE-Q total	1.4 (1.2)	1.3 (1.2)	*t*(141) = 0.42	0.68
BDI-II	13.9 (11.1)	14.9 (11.4)	*t*(141) = −0.51	0.61

## Data Availability

This study’s hypotheses, planned sample, variables, and paradigms were preregistered and can be found on Open Science Framework (https://osf.io/vk7he (accessed on 3 June 2024)), with data and analysis available at https://osf.io/472kr/ (accessed on 3 June 2024).

## Supplementary Materials

The following supporting information can be downloaded at https://www.mdpi.com/article/10.3390/nu16142190/s1, Table S1: Stop-signal task characteristics.
