# Supplementary material for: Modality Matters: Fasted Individuals Inhibit Food Stimuli Better Than Neutral Stimuli for Words, but Not for Pictures
Source: Nutrients. 2024 Jul 9;16(14):2190. doi: 10.3390/nu16142190 (PMC11279540; doi:10.3390/nu16142190)
Supplement: Supplementary file 1 [file nutrients-16-02190-s001.zip › nutrients-3065781-supplementary.pdf]

Table S1: *Stop-signal task characteristics*

	Fasted				Satiated			
	Picture		Word		Picture		Word	
	Food	Non-Food	Food	Non-Food	Food	Non-Food	Food	Non-Food
Go omissions (probability)	0.010	0.011	0.011	0.011	0.010	0.012	0.0080	.008
Go commissions (probability)	0.004	0.004	0.004	0.004	0.003	0.004	0.005	0.004
Stop probability	0.48	0.49	0.49	0.49	0.50	0.50	0.51	0.51
SSD	338	336	335	335	265	276	257	262
SSRT	257	264	255	247	262	257	263	260
RT unsuccessful stop trials	503	491	482	477	493	485	469	470
RT go trials	579	575	562	560	564	557	551	544
SD go trials	143	141	142	143	140	138	141	140

Note: Standard deviation of the go trials reflects reaction time variability. RT = Reaction Time, SSD = Stop-Signal Delay SSRT = Stop-Signal Reaction Time
